# Effects of Curing Regimes on Calcium Oxide–Belite–Calcium Sulfoaluminate-Based Aerated Concrete

**DOI:** 10.3390/ma17194819

**Published:** 2024-09-30

**Authors:** Yanqing Xia, Xirui Lu, Jun Li, Li Yang, Ning Wang, Xuemei Chen, Wen Zhong

**Affiliations:** 1 State Key Laboratory of Environment-Friendly Energy Materials, Southwest University of Science and Technology, Mianyang 621010, China; lijun@swust.edu.cn; 2 JIAHUA Special Cement Co., Ltd., Leshan 614003, China; yangli13880506577@163.com (L.Y.); nwang030206@163.com (N.W.); chunruxue@126.com (X.C.); scjhzw@163.com (W.Z.)

**Keywords:** autoclave–carbonation curing, carbonation degree, microstructure, strength

## Abstract

This study delves into the effects of carbonation curing and autoclave–carbonation curing on the properties of calcium oxide–belite–calcium sulfoaluminate (CBSAC) cementitious material aerated concrete. The objective is to produce aerated concrete that adheres to the strength index in the Chinese standard GB/T 11968 while simultaneously mitigating CO_2_ emissions from cement factories. Results show that the compressive strength of CBSAC aerated concrete with different curing regimes (autoclave curing, carbonation curing, and autoclave–carbonation curing) can reach 4.3, 0.8, and 4.1 MPa, respectively. In autoclave–carbonation curing, delaying CO_2_ injection allows for better CO_2_ diffusion and reaction within the pores, increases the carbonation degree from 19.1% to 55.1%, and the bulk density from 603.7 kg/m^3^ to 640.2 kg/m^3^. Additionally, microstructural analysis reveals that delaying the injection of CO_2_ minimally disrupts internal hydrothermal synthesis, along with the formation of calcium carbonate clusters and needle-like silica gels, leading to a higher pore wall density. The industrial implementation of autoclavecarbonation curing results in CBSAC aerated concrete with a CO_2_ sequestration capacity ranging from 40 to 60 kg/m^3^ and a compressive strength spanning from 3.6 to 4.2 MPa. This innovative approach effectively mitigates the carbon emission pressures faced by CBSAC manufacturers.

## 1. Introduction

Aerated concrete is an advanced material for civil engineering applications with low thermal conductivity (0.07–0.21 W/m·K), low density (400–800 kg/m^3^), good sound insulation ability, and good fire resistance [1,2,3]. It is extensively utilized in the walls, roofing, and external wall cladding of buildings in cold regions to reduce energy consumption and CO_2_ emissions [4,5,6].

Autoclaved aerated concrete (AAC) is typically produced by autoclaving calcareous and siliceous materials, wherein ordinary Portland cement (OPC) and quicklime are the primary calcium sources [7,8]. In the AAC production process, it is essential to simultaneously control the quality and ratio of OPC and quicklime to ensure that the final products meet the required standards. To simplify the AAC production process, the calcium oxide–belite–calcium sulfoaluminate (CBSAC) cementitious material has been developed as an effective substitute for OPC and lime. CBSAC has been successfully used in AAC production, with the resulting product meeting the quality requirements of Chinese standard GB/T 11968 [9]. However, the impact of other curing methods on the performance of CBSAC aerated concrete has yet to be explored.

Additionally, calculations reveal that carbon emissions from carbonate decomposition in CBSAC production range from 0.54 to 0.61 tons per ton of CBSAC, while those from carbonate decomposition in OPC clinker production range from 0.48 to 0.54 tons of CO_2_ per ton of OPC clinker. Thus, CBSAC exhibits a higher carbon emission level. In light of China’s “dual carbon” targets and the planned inclusion of cement in the carbon trading market by 2025, cement factories producing CBSAC will face considerable environmental pressures. Therefore, reducing CO_2_ emissions from these factories is critical.

Recently, flue gas carbonation has been successfully used to cure building products for CO_2_ capture and storage [10,11]. Carbonation curing can be used to accelerate the hydration of cementitious materials and improve the performance of concrete products at an early age [12]. Two approaches are available to early-age carbonation curing of concrete mixtures. If the concrete mixture is cured after casting, the binder minerals (e.g., C_2_S, C_3_S, and f-CaO) react with CO_2_ as follows [13,14]:

(1) $$C_{3}S+(3-x)CO_{2}+yH_{2}O\to (3-x)CaCO_{3}+C_{x}SH_{y}$$

(2) $$C_{2}S+(2-x)CO_{2}+yH_{2}O\to (2-x)CaCO_{3}+C_{x}SH_{y}$$

(3) $$f-CaO+CO_{2}+2H_{2}O\to CaCO_{3}+2H_{2}O.$$

If the concrete mixture is cured after a short period of initial hydration, anhydrous binder minerals (e.g., C_2_S, C_3_S, and f-CaO) and hydration products (e.g., Ca(OH)_2_ and C–S–H) undergo carbonation, and the following additional reactions occur [15]:

(4) $$Ca{\left(OH\right)}_{2}+CO_{2}\to CaCO_{3}+H_{2}O$$

(5) $$C-S-H+CO_{2}\to CaCO_{3}+SiO_{2}+H_{2}O$$

Several studies have examined the factors that influence early-age carbonation curing of concrete. Optimal moisture content in concrete allows for the highest degree of carbonation, which may vary depending on the water-to-binder ratio (w/b ratio) [16]. Additionally, CO_2_ concentration [17] and pressure [18], curing temperature [19], and humidity [17], directly influenced the CO_2_ diffusivity. Moreover, concrete’s porosity and pore structure [18] are also crucial factors in carbonation curing.

Compared with ordinary concrete, aerated concrete has a higher porosity, which enhances CO_2_ diffusion in its pores and allows for a higher CO_2_ storage rate even at lower CO_2_ partial pressures [20]. In recent years, the use of carbonation curing for aerated concrete has gradually increased. The authors of [21] reported the traditional autoclaving process was augmented with a carbonation stage to produce steel slag-based autoclaved aerated concrete. After 12 h of carbonation-autoclaving curing, the strength of the aerated concrete ranges from 1.1 to 2.1 MPa, with a bulk density of 490 kg/m^3^. Chang [22] reported on a CO_2_-cured aerated concrete made from steel slag, with a sample density of 590 kg/m^3^ and a compressive strength of 1.0 MPa. The strength of the aerated concrete in the aforementioned literature falls short of the strength requirements set by the Chinese standard GB/T 11968.

This study delves into carbonation curing and autoclave–carbonation curing of CBSAC aerated concrete, intending to fulfill the strength specifications outlined in the Chinese standard GB/T 11968, concurrently mitigating the release of CO_2_ emissions from cement factories. First, within a laboratory setting, the impact of the carbonation regime on various aspects, including the physical and mechanical properties, carbonation degree, pH value, hydration products, and pore characteristics, were investigated in comparison to autoclave curing. Furthermore, based on the existing equipment conditions of a rotary cement kiln, a 10 m^3^ autoclave container was used to capture flue gas and superheated steam from waste heat boilers for conducting autoclave–carbonation curing of CBSAC aerated concrete, presenting innovative perspectives for cement enterprises to enhance CO_2_ sequestration.

## 2. Materials and Methods

### 2.1. Raw Materials

The raw materials comprised CBSAC as the calcareous material, red sand powder as the siliceous material, gypsum as the auxiliary material, and Al paste as the gas-generating agent.

CBSAC was provided by Jiahua Special Cement Co., Ltd., and it was produced by sintering limestone, sand, gypsum, and bauxite in a rotary kiln at 1250–1320 °C [9,23]. Table 1 presents the physical properties of CBSAC.

Red sand and gypsum originated in Leshan in Sichuan Province, China. The red sand was finely pulverized in a ball mill (SM-500, Jianyi, Wuxi, China. Cylinder size: ψ 500 × 500 mm, rotational speed: 48 r/min, steel ball loading capacity: 100 kg, grinding time: 25 min), and it had a 45 µm screen residue weight of 31.8%.

The 45 µm screen residue weight of gypsum was 22.1%, while its water crystallization content was 20.3%. In the autoclave curing stage, gypsum facilitates the conversion of C–S–H into tobermorite and promotes the incorporation of aluminum ions into tobermorite, which inhibits the formation of hydrated garnet [24,25].

The commercially available aluminum paste from Shangdong Juxing Chemical Co., Ltd. in China, with an effective aluminum content of 75%, reacts with Ca(OH)_2_ to generate H_2_, resulting in the formation of bubbles.

**Figure 1:**
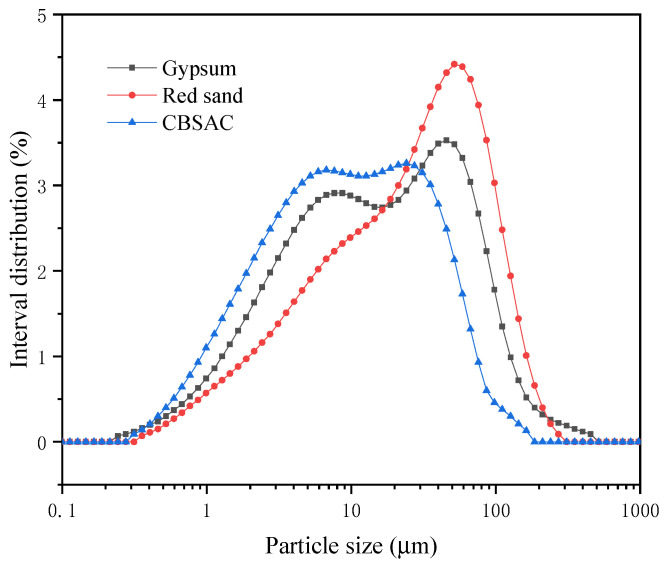
Particle size distribution of raw materials.

Table 2 presents the chemical composition of raw materials. Figure 1 and Table 3 present the particle size distribution of raw materials.

### 2.2. Sample Preparation

CBSAC aerated concrete samples were prepared via a three-step process: mixing and agitation, foaming and pre-curing, and curing.

#### 2.2.1. Mixture Proportion and Agitation Process

CBSAC, red sand powder, and gypsum were mixed at constant proportions of 38%, 57%, and 5%, respectively, based on many previous experiments. The Al paste and water accounted for 0.12% and 67%, respectively, of the total mass of dry ingredients. Dry ingredients weighing 3 kg were mixed in a laboratory mixer for 1 min. Subsequently, the mixture was supplemented with 85% water and rapidly mixed for another 1 min. The remaining water was then used to disperse 3.6 g of the Al paste into the mixture, which was mixed again for 30 s. Finally, the resulting slurry was poured into cubic molds that were 100 mm in size.

#### 2.2.2. Foaming and Pre-Curing

After the slurry was poured into the molds, the molds were placed into a steam-curing box at 50 °C. The slurry was subjected to a foaming process with a standing time of 45 min, followed by pre-curing for 3 h. Subsequently, the excess mortar that protruded from the molds was carefully removed before demolding.

#### 2.2.3. Curing Method

**Figure 2:**
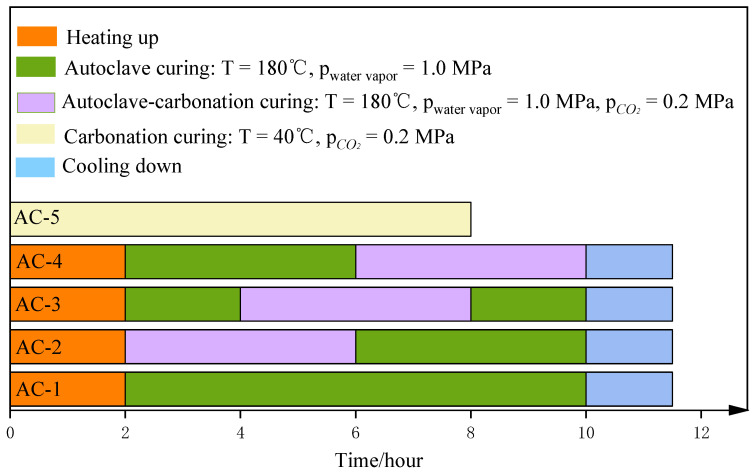
Curing regimes.

Commercial AAC is cured in an autoclave environment at a temperature of 180–200 °C and pressure of 1.0–1.5 MPa for 5–8 h to produce tobermorite [26,27,28,29]. In this study, the molds were removed after the pre-curing stage to obtain green bodies. The green bodies were placed in a self-developed autoclave, and the various curing regimes shown in Figure 2 were applied to obtain the final CBSAC aerated concrete samples. AC-1 corresponded to autoclave curing only as a control group. AC-2, AC-3, and AC-4 corresponded to different combinations of autoclave–carbonation curing. AC-5 corresponded to carbonation curing only.

Each sample ID was subjected to three parallel curing procedures, with six specimens per curing procedure, resulting in a total of 18 specimens per sample ID.

#### 2.2.4. Sampling Position

**Figure 3:**
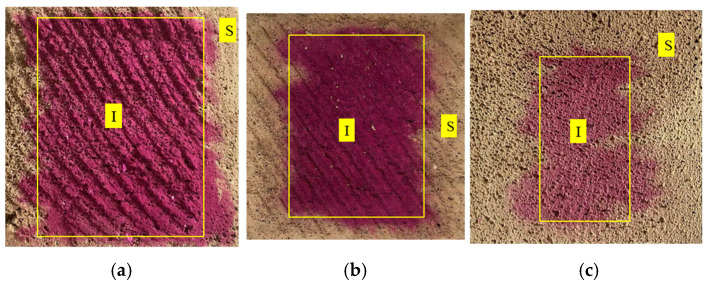
Sampling positions with the autoclave–carbonation curing regimes (I: Interior and S: Surface). (**a**) AC-2, (**b**) AC-3, (**c**) AC-4.

The pH value and microstructure of the AC-2, AC-3, and AC-4 samples were measured at the interior (I) and surface (S), respectively (Figure 3).

### 2.3. Testing and Analysis Method

#### 2.3.1. Bulk Density and Compressive Strength

The bulk density and compressive strength of the CBSAC aerated concrete samples were determined by following the Chinese standard GB/T 11969-2020 [30]. To determine the bulk density, 10 cm × 10 cm × 10 cm samples were dried in a ventilated oven at 60 ± 5 °C for 24 h. Subsequently, the samples were dried at 80 ± 5 °C for another 24 h and further dried at 100 ± 5 °C to a constant weight (M_0_). The bulk density was determined by dividing M_0_ by the volume (1000 cm^3^). Each dried sample was positioned at the center of the press machine and subjected to a continuous and uniform loading speed of (2.0 ± 0.5) kN/s until failure occurred. The breaking load (P) was recorded, and the compressive strength (σ) was calculated using the relation σ (MPa) = P (N)/A (mm^2^). Average bulk density and compressive strength were calculated from a random sample of nine cubes, plotting the picture using “mean ± standard deviation”.

#### 2.3.2. Carbonation Degree Tests

The cubic samples were split in the middle, and the phenolphthalein indicator was sprayed on the fracture surface after curing. The ratio of the uncolored area to the total cross-sectional area of a sample was utilized to determine the carbonation degree. The uncolored area was determined using a convolutional neural network-based image segmentation technique [31]. The average carbonation degree from a random sample of 3 cubes was calculated.

#### 2.3.3. pH Value Tests

The pH value of aerated concrete was determined through solid-liquid extraction. First, the dried cubic samples were ground using a disk attrition mill (SKZM-1, Shengke Instrument, Shaoxing, China), and the powder sample was sieved through an 80 µm sieve. Subsequently, 10 g of the powder that passed through the sieve was dispersed in 100 g of distilled water. Finally, the solution was filtered and measured using a digital pH meter (PHS-3E, INESA Instrument, Shanghai, China) within a controlled environment of 25 ± 1 °C.

#### 2.3.4. Characterization

The chemical composition of the raw materials was determined using an X-ray fluorescence spectrometer (Axios X, Malvern Panalytical, Malvern, UK; The measured elements span from fluorine to uranium, relative standard deviation: 0.1–1%, positioning accuracy: 0.00025°).

The mineral composition of the aerated concrete products was analyzed via XRD (D8 Advance, Bruker, Billerica, MA, USA) using Cu Kα radiation (λ = 1.5406 Å) at 60 kV and 80 mA. The powders were scanned in the range of 2θ = 5–70° at a step size of 0.02° and scanning time of 0.5 s per step.

The morphologies of products were investigated via scanning electron microscopy (SEM; Quattro, Thermo Scientific, Waltham, MA, USA), while their composition was analyzed via energy-dispersive spectroscopy (EDS; Emax50, Horiba, Kyoto, Japan). The parameters used for SEM and EDS measurements were as follows: resolution: 1.0 nm, accelerating Voltage: 0.1 kV to 30 kV, magnification: 1000×–8000×, and sample diameter: 2–5 mm.

The crystal properties of the products were analyzed via ^29^Si nuclear magnetic resonance (^29^Si NMR; Advance 600, Bruker, Fällanden, Switzerland) spectroscopy. The magnetic field strength of the spectrometer was set at 9.40 T, and the resonance frequency for ^29^Si was 79.49 MHz. Moreover, the samples used ^29^Si NMR spectroscopy were in powder form.

The nitrogen adsorption technique (Autosorb-1, Malvern, UK) was employed to characterize the microscopic pore structure of the aerated concrete samples. The samples were degassed at 35 °C for 6 h before the test. The nitrogen adsorption test was conducted at 77.35 K and a relative pressure (p/p_0_) of 10^−2^–1.0.

## 3. Results and Discussion

### 3.1. Hydration Mechanism of CBSAC

**Figure 4:**
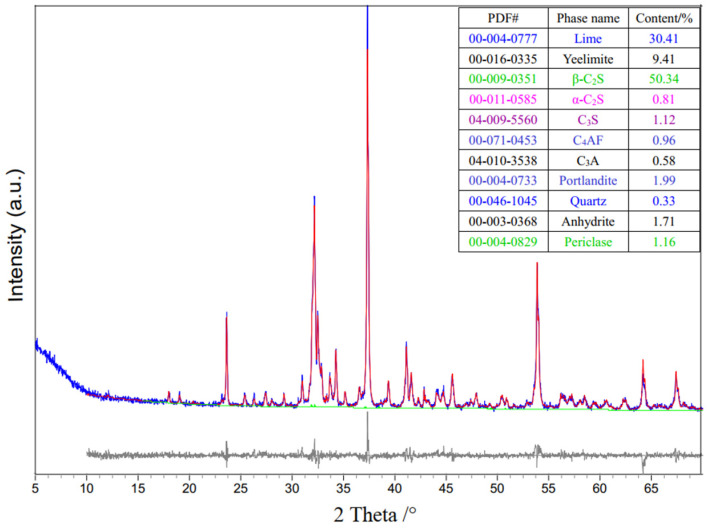
Rietveld quantitative phase analysis of CBSAC.

**Figure 5:**
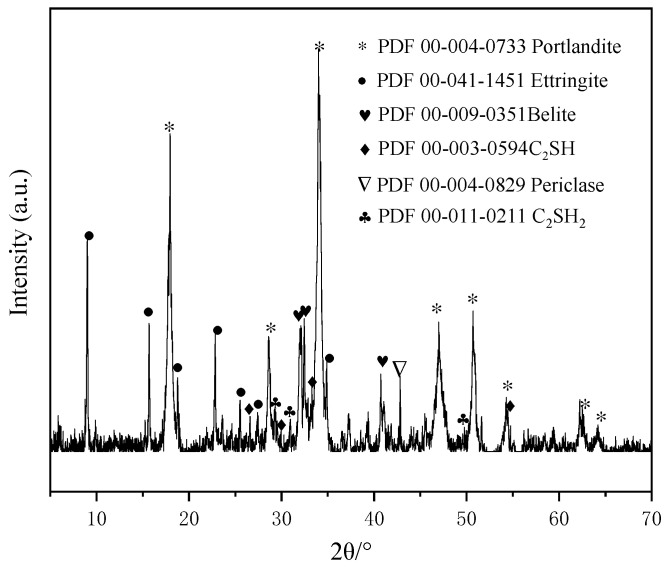
XRD pattern of CBSAC paste hydrates in the pre-curing stage.

Figure 4 presents the direct Rietveld quantitative phase analysis findings of CBSAC, featuring an Rwp value of 8.5 (the Rwp value serves as a crucial criterion for assessing the quality of the XRD pattern fit, and it should remain below 15.0), with the crystalline phases normalized to 100%. Table 4 presents the main mineral content of CBSAC from the direct Rietveld quantitative phase analysis. Figure 5 shows the XRD pattern of CBSAC paste hydrates in the pre-curing stage. As shown in Figure 4 and Table 4, the main minerals in CBSAC were 30.4% CaO, 51.2% C_2_S, and 9.4% C_4_A_3_$\bar{S}$. As shown in Figure 5, CaO, C_4_A_3_$\bar{S}$, C_2_S and other minerals of CBSAC react with water and gypsum to generate Ca(OH)_2_, AFt, the high-alkalinity C–S–H (C_2_SH and C_2_SH_2_). The reaction processes are as follows:

(6) $$CaO+H_{2}O\to Ca{\left(OH\right)}_{2}$$

(7) $$C_{4}A_{3}\bar{S}+2CaSO_{4}\cdot 2H_{2}O+34H_{2}O\to AFt+2Al{\left(OH\right)}_{3}$$

(8) $$C_{2}S+nH_{2}O\to C_{2}SH_{n}\;(n=1,2)$$

The reaction between CaO and water can enhance the alkalinity of the aerated concrete slurry, creating an alkaline environment for Equation (9) and promoting the gas-producing reaction of aluminum paste.

(9) $$2Al+2OH^{-}+6H_{2}O\to 2{\left[Al{\left(OH\right)}_{4}\right]}^{-}+3H_{2}↑$$

The hydration of CaO is a highly exothermic reaction, providing a substantial amount of heat to facilitate further condensation and solidification. Furthermore, AFt and the high-alkalinity C–S–H contribute to the initial consistency of the slurry and thereby stabilize bubbles while promoting thickening and hardening, which facilitates the formation of a uniform porous structure.

### 3.2. Physical and Mechanical Properties

**Figure 6:**
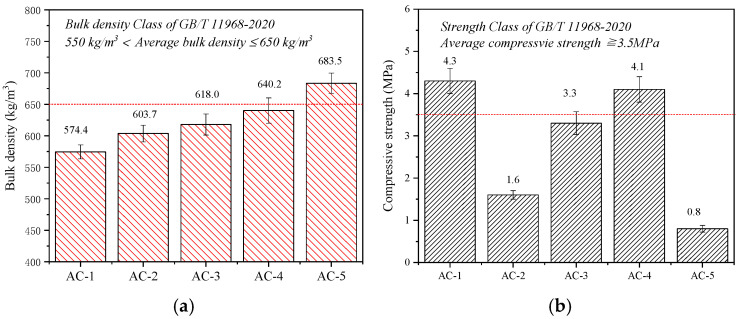
Physical and mechan ical properties of samples: (**a**) Bulk density, (**b**) Compressive strength.

The physical and mechanical properties of AC-1, AC-2, AC-3, AC-4, and AC-5 are presented in Figure 6. Figure 6a shows that the bulk density is lowest for autoclave curing only (AC-1) at 574.4 kg/m^3^. Autoclave–carbonation curing increases the bulk density to 603.7 (AC-2), 618 (AC-3), and 640.2 (AC-4) kg/m^3^. Autoclave–carbonation curing led to a bulk density increase of 29~66 kg/m^3^ compared to autoclaved curing. Moreover, carbonation curing only (AC-5) considerably increases the bulk density to 683.5 kg/m^3^. These results indicate that applying carbonation curing requires green bodies with a lower bulk density than those required for applying autoclave curing.

Furthermore, Figure 6b shows that the compressive strengths of the CBSAC samples considerably vary with different curing regimes. AC-1 exhibits the highest compressive strength at 4.3 MPa. Moreover, the compressive strengths for the autoclave–carbonation curing regimes increase from 1.6 MPa for AC-2 to 4.1 MPa for AC-4, and the bulk density and strength of AC-4 meet the index requirements of B_06_A_3.5_ in Chinese standard GB/T11968 [32]. Notably, delaying CO_2_ injection into the autoclave during autoclave–carbonation curing mitigated its impact on compressive strength. AC-5, cured solely through carbonation, demonstrated a markedly lower compressive strength of 0.8 MPa, falling short of the strengths achieved by other curing methods. The curing regime considerably affects the hydration products, which makes it a critical factor that determines the physical and mechanical properties of CBSAC aerated concrete. These results will be discussed further in Section 3.5 in the context of more characterization methods.

### 3.3. Carbonation Degree

**Figure 7:**
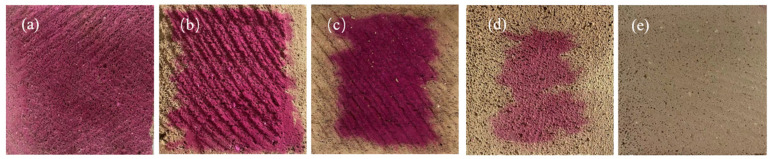
Color reaction of phenolphthalein in samples according to the curing regime: (**a**) AC-1, (**b**) AC-2, (**c**) AC-3, (**d**) AC-4, and (**e**) AC-5.

Figure 7 and Table 5 present the carbonation degrees of the samples after curing. The carbonation degrees of AC-1, AC-2, AC-3, AC-4, and AC-5 are 0.0%, 19.1%, 36.8%, 55.1%, and 100%, respectively. Among the autoclave–carbonation curing regimes (i.e., AC-2, AC-3, and AC-4), introducing CO_2_ later results in a greater carbonation degree. Autoclave curing comprises three stages: heating, constant temperature, and cooling. During the heating stage, the saturated water vapor penetrates the green body through capillaries to release heat, increase the internal temperature, and condense into the water in the pores, which results in rapid humidification of the green body and increased bulk density [33,34]. Thus, the internal pores of the aerated concrete are saturated with condensed water at the beginning of the constant-temperature stage, which obstructs the diffusion of CO_2_ to the reactants. However, the water evaporation and condensation in aerated concrete gradually reach equilibrium after 2–4 h of the constant-temperature stage, which reduces the water content within the pores [34]. This facilitates the diffusion of CO_2_ to the reactants in AC-3 and AC-4.

### 3.4. pH Value

Table 6 presents the pH values of the samples after different curing regimes. AC-1 exhibits a pH value of 11.07, which is less than that of ordinary concrete (pH > 12). This can be attributed to Ca(OH)_2_ and high-alkalinity C_2_SH or C_2_SH_2_ reacting with quartz to form tobermorite [35,36]. For the autoclave–carbonation-cured samples, the pH values are lower at the surface (S) than at the interior (I). The pH values of AC-2 (S), AC-3 (S), and AC-4 (S) are 9.28, 9.24, and 9.16, respectively, while the pH values of AC-2 (I), AC-3 (I), and AC-4 (I) are 11.09, 11.05, and 11.00, respectively. In addition, AC-5 has a pH value of 8.86, which indicates that carbonation curing decreases the pH value.

Compared with the pH value of AC-1, the pH value of AC-2 (I) increases slightly, whereas the pH values of AC-3 (I) and AC-4 (I) decrease slightly. The pH values of AC-2 (S), AC-3 (S), and AC-4 (S) are slightly higher than the pH value of AC-5, owing to the incomplete carbonation reaction.

### 3.5. Microstructure

#### 3.5.1. XRD

**Figure 8:**
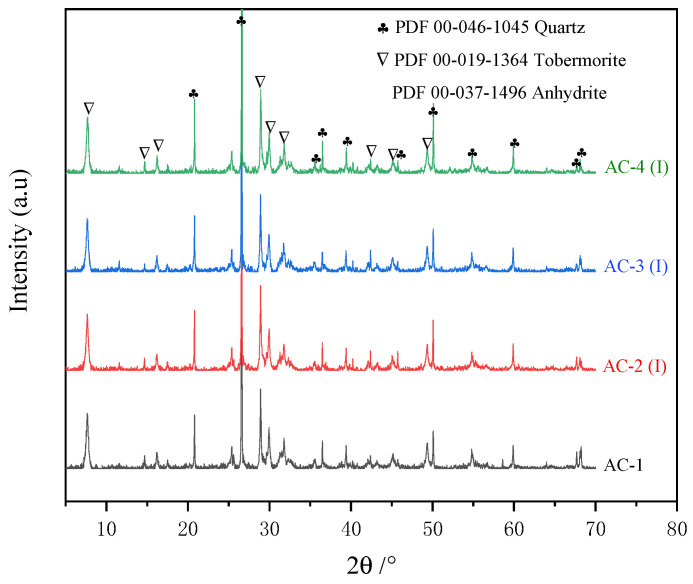
XRD patterns of AC-1, AC-2 (I), AC-3 (I), and AC-4 (I).

Figure 8 shows the diffraction patterns of AC-1, AC-2 (I), AC-3 (I), and AC-4 (I). The crystalline phases of AC-1 mainly comprise tobermorite, anhydrite, and residual quartz from red sand. In the pre-curing stage, the reaction between gypsum and C_4_A_3_$\bar{S}\;$produces AFt in the slurry, which facilitates solidification. However, AFt decomposition produces anhydrite during autoclave curing [4]. The SiO_2_ in red sand consumes Ca(OH)_2_, C_2_SH, and C_2_SH_2_ to form tobermorite through a hydrothermal reaction. AC-2 (I), AC-3 (I), and AC-4 (I) exhibit crystalline phases such as quartz, anhydrite, and tobermorite, which are consistent with those found in AC-1. These results indicate that the hydrothermal reaction occurs within the interior of the autoclave–carbonation–cured samples.

**Figure 9:**
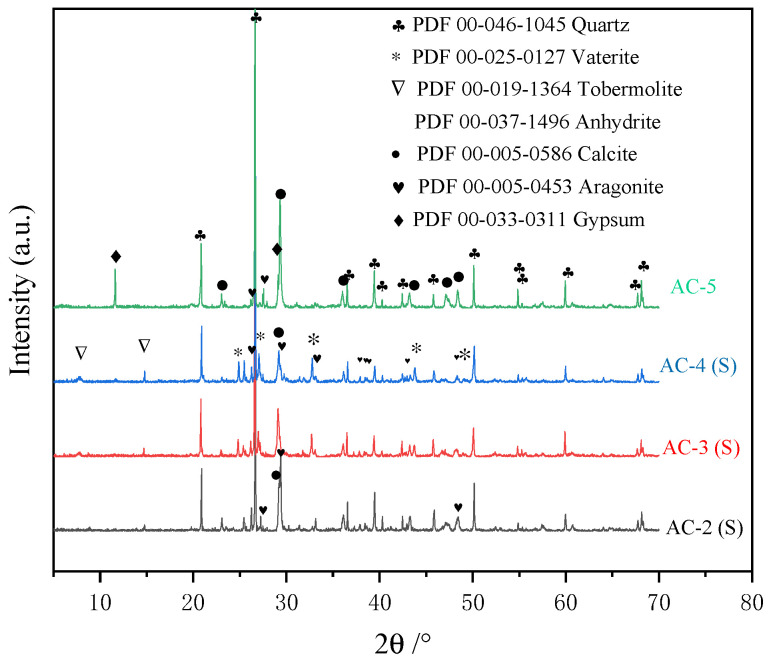
XRD patterns of AC-2 (S), AC-3 (S), AC-4 (S), and AC-5.

Figure 9 shows the XRD patterns of AC-2 (S), AC-3 (S), AC-4 (S), and AC-5. The crystalline phases in AC-2 (S), AC-3 (S), and AC-4 (S) comprise quartz, anhydrite, and calcium carbonate. Calcium carbonate is a product of the reaction between the hydration products and CO_2_. Peaks associated with calcite and aragonite are observed for AC-2 (S), AC-3 (S), and AC-4 (S), and the intensity of the calcite diffraction peak gradually diminishes as CO_2_ is introduced later. Moreover, AC-3 (S) and AC-4 (S) exhibit signals at 24.8°, 27.2°, and 32.8°, indicating the presence of vaterite coexisting with calcite and aragonite as crystalline forms of calcium carbonate. In addition, the peaks of tobermorite are observed at varying degrees in AC-3 (S) and AC-4 (S). The crystalline phases of AC-5 mainly comprise calcite, aragonite, quartz, and gypsum. Gypsum is associated with the carbonation of AFt. Calcium carbonate exists as calcite and aragonite in AC-5.

#### 3.5.2. SEM-EDS

**Figure 10:**
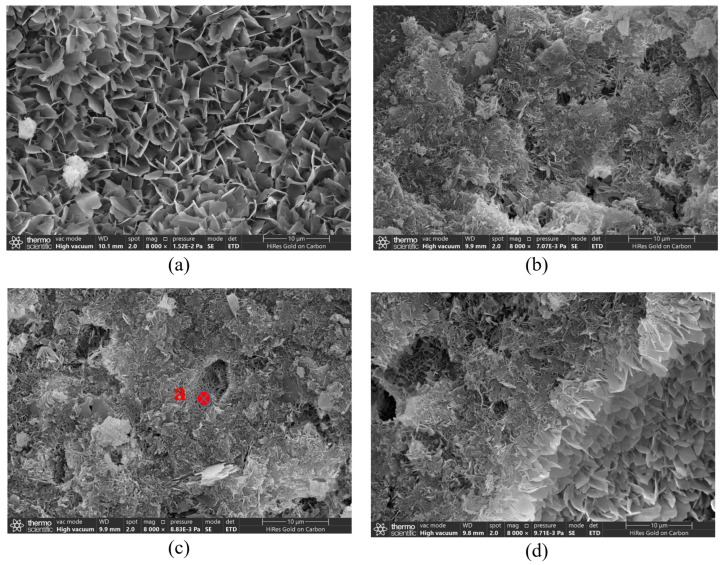

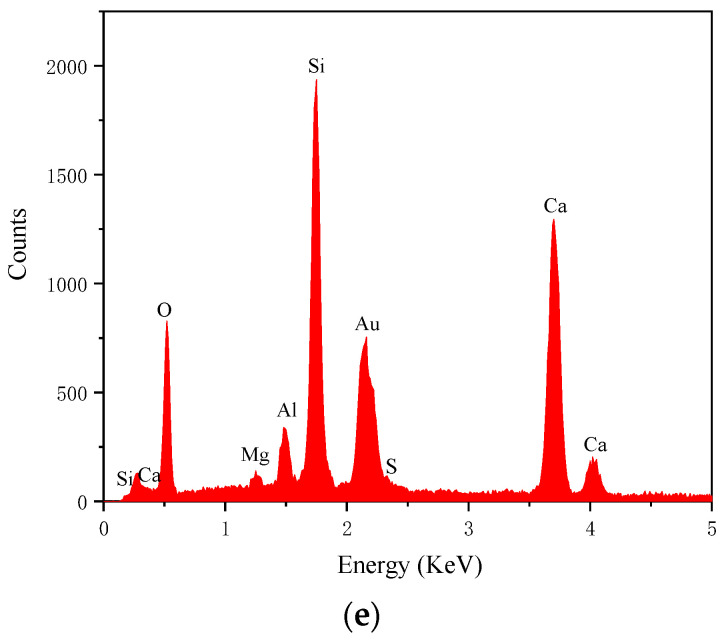
Microstructure of samples: (**a**) AC-1, (**b**) AC-2 (I), (**c**) AC-3 (I), (**d**) AC-4 (I), and (**e**) EDS analysis of site a.

Figure 10 presents SEM-EDS images of the microstructure of AC-1, AC-2 (I), AC-3 (I), and AC-4 (I). As shown in Figure 10a–d, different morphologies of products could be observed in the samples. According to the literature [37], the plate-like crystal in Figure 10a is tobermorite. Figure 10b,c illustrate that the pore walls of AC-2 (I) and AC-3 (I) consist of needle-like crystals. Furthermore, the EDS analysis depicted in Figure 10e and the XRD patterns of AC-3 (I) verify that these needle-like crystals are tobermorite. Figure 10d shows AC-4 (I) has needle-like and plate-like tobermorite. For autoclave–carbonation curing, the SEM-EDS results show that injecting CO_2_ into the autoclave later causes carbonation to exert less effect on the production of plate-like tobermorite in the interior, which potentially contributes to the increase in compressive strength.

**Figure 11:**
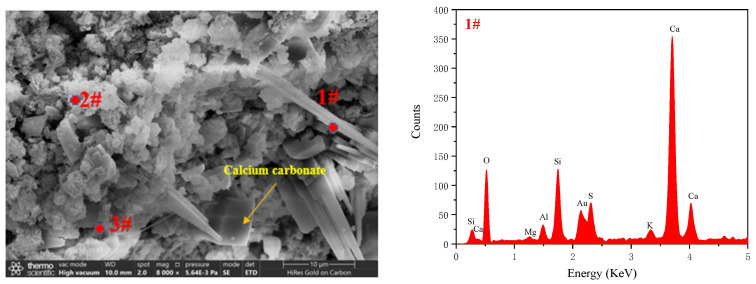

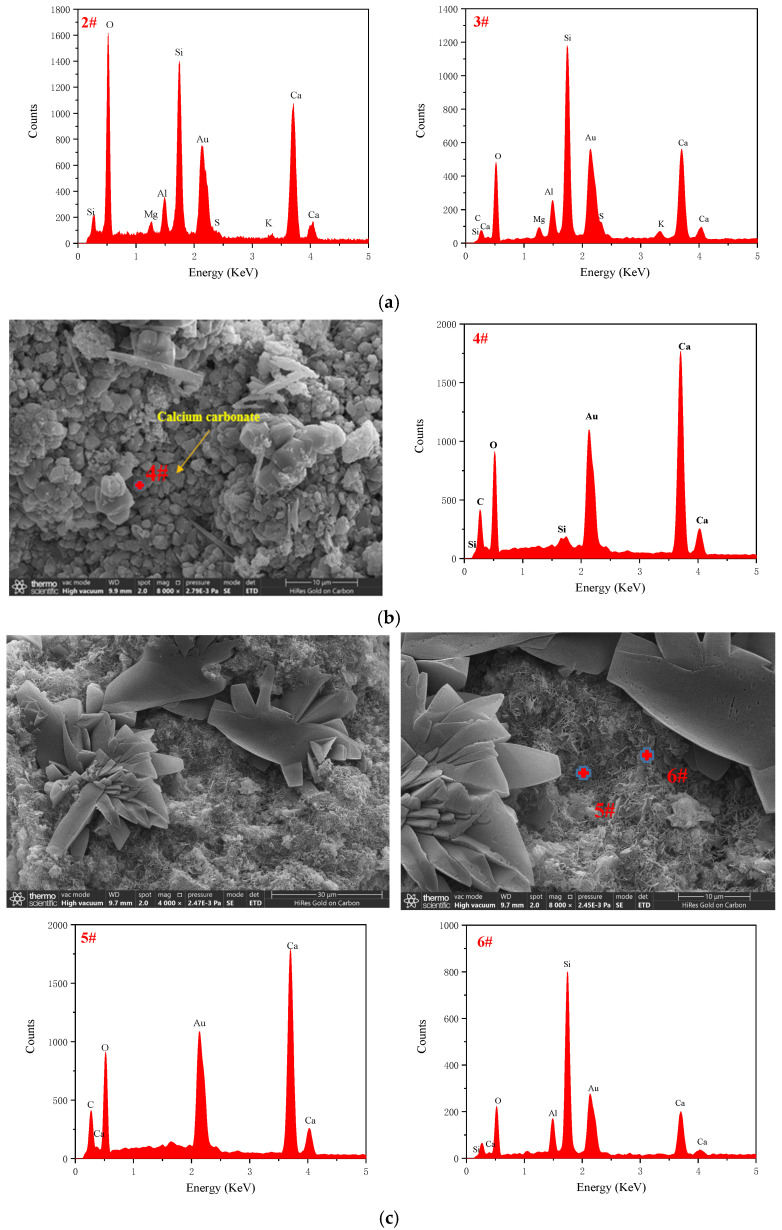

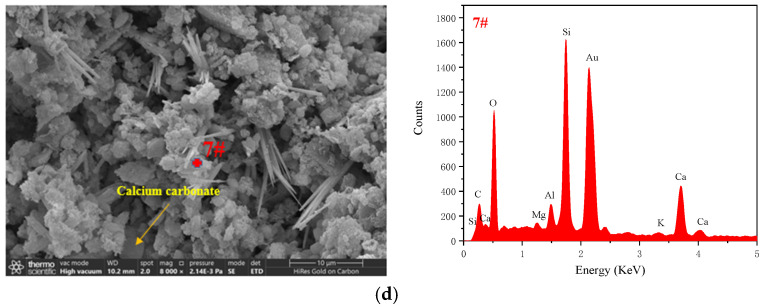
Microstructure of samples: (**a**) AC-2 (S), (**b**) AC-3 (S), (**c**) AC-4 (S), and (**d**) AC-5.

Figure 11 shows microstructure images of AC-2 (S), AC-3 (S), AC-4 (S), and AC-5. Among the autoclave–carbonation curing regimes, the pore wall morphologies of AC-2 (S), AC-3 (S), and AC-4 (S) differ substantially in carbonated regions. As shown in Figure 11a, EDS analysis of sites #1 and #2 indicates that the pore walls of AC-2 (S) have a large accumulation of floccule and needle-like C–S–H along with cube-shaped calcium carbonates. The strong peaks of C, O, Si, and Ca for site #3 confirm that floccule C–S–H wraps calcium carbonates to produce composite products of cementitious matrix. Figure 11b shows that the pore walls of AC-3 (S) have many cube-shaped calcium carbonate crystals along with minor amounts of C–S–H. Figure 11c shows the SEM and EDS analysis results for site #5, which indicate that clump-like calcium carbonates are present on the surface of the pore walls. The strong peaks of Si for site #6 confirm the presence of needle-like crystals corresponding to SiO_2_ gel, which can be attributed to the “pseudomorphism” of decalcified tobermorite [38,39]. In comparison, carbonation curing occurs at a considerably lower temperature of 40 °C, necessitating more time for the intermingling of C–S–H and calcium carbonates, as inferred from the C peak of C–S–H for Site #7 (Figure 11d).

#### 3.5.3. ^29^Si Nuclear Magnetic Resonance Spectroscopy

**Figure 12:**
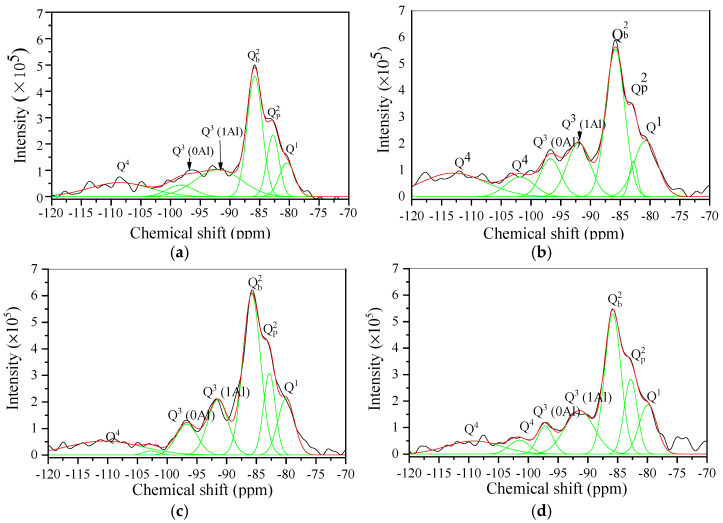
^29^Si NMR results of samples with autoclave curing: (**a**) AC-1, (**b**) AC-2 (I), (**c**) AC-3 (I), and (**d**) AC-4 (I). The fits and deconvoluted peaks for the spectra of the samples are shown as red and green lines, respectively.

**Figure 13:**
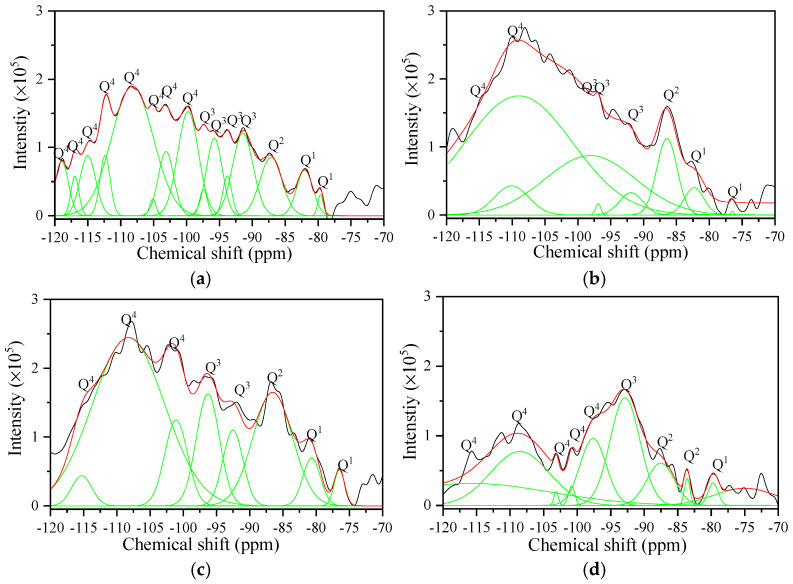
^29^Si NMR results of samples with carbonation curing: (**a**) AC-2 (S), (**b**) AC-3 (S), (**c**) AC-4 (S), and (**d**) AC-5. The fits and deconvoluted peaks for the spectra of the samples are shown as red and green lines, respectively.

Figure 12 and Figure 13 present the ^29^Si NMR results of all samples, while Table 7 and Table 8 present the Q^1^, Q^2^, Q^3^, and Q^4^ values obtained from Gaussian fitting. ^29^Si NMR was used to characterize the chemical environment of Si in the aerated concrete samples, which is typically represented by Q^n^ [40], where n denotes the quantity of bridged oxygen (i.e., oxygen atoms connecting two silicon-oxygen tetrahedrons). The central peaks of the chemical shifts range from −76 to 82 ppm for Q^1^ (i.e., dimer and chain end groups), −82 to −88 ppm for Q^2^ (i.e., chain middle groups), −88 to −98 ppm for Q^3^ (i.e., layers and chain branching sites), and −98 to −129 ppm for Q^4^ (i.e., three-dimensional crosslinked frameworks) [41].

In AC-1, AC-2 (I), AC-3 (I), and AC-4 (I), the hydrothermal reaction of calcareous and siliceous materials generated tobermorite. As presented in Figure 12 and Table 7, these samples exhibit signals at 80.1, 82.8, 85.8, 92.0, 97.1, and 110 ppm corresponding to Q^1^, $Q_{P}^{2}$(1Al), $Q_{\;b}^{2}$, Q^3^ (1Al), Q^3^ (0Al), and Q^4^, respectively [42,43]. The mean chain length (MCL) of tobermorite is calculated as follows [44]:

(10) $$\mathrm{MCL}=\frac{4\left[Q^{1}{+Q}_{P}^{2}{(\mathrm{Al})+Q}_{b}^{2}{+Q}^{3}{+2Q}^{3}\left(\mathrm{Al}\right)\right]}{Q^{1}}$$

The tobermorite in AC-1 has an MCL of 40.6, which is greater than the MCLs of AC-2 (I), AC-3 (I), and AC-4 (I) at 23.2, 27.6, and 31.8, respectively. These results indicate that tobermorite has a lower degree of polymerization with autoclave–carbonation curing than with autoclave curing alone. The MCLs of tobermorite in AC-2 (I), AC-3 (I), and AC-4 (I) also indicate a direct correlation between the degree of polymerization and when CO_2_ is injected into the autoclave. It is not difficult to find that the levels of tobermorite polymerization (Table 7) are consistent with the compressive strength development (Figure 6b).

As presented in Figure 13 and Table 8, the predominant structure in AC-2 (S), AC-3 (S), AC-4 (S), and AC-5 is SiO_4_ tetrahedra with Q^4^ values of 66.5%, 66.4%, 59.6%, and 48.5%, respectively, and Q^3^ values of 22.7%, 23.8%, 16.9%, and 37.9%, respectively. Q^3^ and Q^4^ represent the SiO_4_ tetrahedra on the surface and in the interior, respectively, and these values indicate that the siliceous product (i.e., silica gel) of the carbonation reaction is a three-dimensional network structure of interconnected SiO_4_ tetrahedra. The degree of polymerization of the silica gel can be calculated from the ratio of Q^4^/Q^3^ [45]. The silica gel in AC-5 has a Q^4^/Q^3^ value of 1.28, which is lower than the Q^4^/Q^3^ values of 2.94, 2.78, and 3.57 for AC-2 (S), AC-3 (S), and AC-4 (S), respectively. These results indicate that the silica gel has a higher degree of polymerization in samples with autoclave–carbonation curing than with carbonation curing only.

#### 3.5.4. Pore Structures

**Figure 14:**
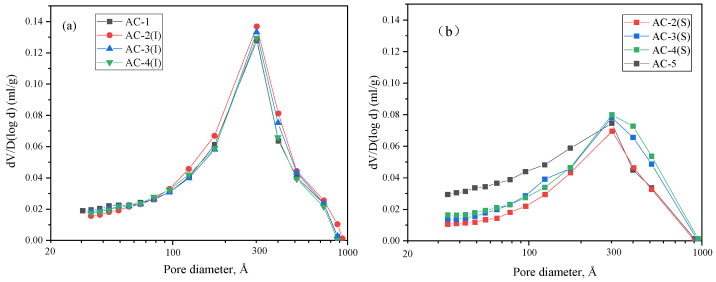
Pore size distributions of samples according to the curing regime: (**a**) AC-1, AC-2 (I), AC-3 (I), and AC-4 (I); (**b**) AC-2 (S), AC-3 (S), AC-4 (S), and AC-5.

Figure 14 shows the micropore distribution according to the nitrogen adsorption isotherm. The pores in the aerated concrete samples were classified according to their size: air pores introduced by the aerating agent had a diameter of 100–1000 µm, microcapillaries had a diameter of 100 nm–100 µm, and micropores had a diameter of 100 nm or less [2]. As shown in Figure 14a, AC-2 (I) has a slightly greater pore volume than the other three samples in the range of 100–1000 Å. As shown in Figure 14b, AC-5 has a greater micropore volume than the other three samples in the range of 30–300 Å; however, AC-4 (S) has the largest micropore volume in the range of 300–1000 Å. In addition, AC-2, AC-3, and AC-4 exhibit a smaller number of micropores at the surface (Figure 14b) than in the interior (Figure 14a) in the range of 30–1000 Å. The results show that carbonation can reduce the content of micropores.

### 3.6. Discussion

The XRD, SEM, and ^29^Si NMR results for AC-1 reveal that the high-alkalinity C–S–H, Ca(OH)_2_, AFt, and unhydrated CBSAC particles react with quartz during autoclave curing to form highly polymerized plate-like tobermorite. The morphologies of the tobermorite align with previous research findings [46,47,48,49]. The pore walls of AC-1 are interconnected by the tobermorite, which results in a dense microstructure and a strength of 4.3 MPa.

The XRD and SEM analyses of AC-5 samples reveal that the resulting products from the carbonation curing process comprised calcite, aragonite, C–S–H, and gypsum. These findings are in agreement with previous studies on carbonation-cured cement paste [41,50,51]. Placing the green body immediately in contact with CO_2_ facilitates the carbonation of C_2_S, Ca(OH)_2_, high-alkalinity C–S–H, and AFt. The chemical reactions of the first three are presented in Equations (2), (4), and (5), while the chemical reaction of the last is given below [15,52]:

(11) $$AFt+3CO_{2}\overset{H_{2}O}{\to }3CaSO_{4}\cdot 2H_{2}O+3CaCO_{3}+Al_{2}O_{3}\cdot xH_{2}O+(26-x)H_{2}O$$

Samples with carbonation curing exhibit a smaller micropore size than samples with autoclave curing (Figure 14), which contributes to the performance of carbonated aerated concrete. In addition, some calcium carbonate crystals and floccule C–S–H are closely intermingled to generate a hybrid of hydrates and carbonates. Gu et al. [21] reports that silica gel and C–S–H coat the surface of crystalline CaCO_3_ to form a hybrid, contributing to compressive strength growth. However, the hybrid has limited content, and it cannot effectively bond with sand particles to form a strong pore wall structure (Figure 11d). Consequently, AC-5 exhibits a compressive strength of only 0.8 MPa, indicating that carbonation-cured CBSAC aerated concrete falls short of the application requirements despite its carbonation degree reaching 100%.

Figure 6b and Figure 7 show that delaying the CO_2_ injection increases the strength and carbonation degree of the autoclave–carbonation-cured aerated concrete. At the beginning of the constant temperature stage, the carbonation reaction for AC-2 is hindered by excessive water content, which results in a carbonation degree of only 19.1% for AC-2 (Table 6), and its performance is mainly determined by the characteristics of the autoclaved products. Thanks to CO_2_ having a lower thermal conductivity than that of water vapor [53,54], injecting CO_2_ into the autoclave reduces the heat transfer efficiency of the medium and then reduces levels of tobermorite polymerization (Table 7) in the AC-2 interior zone. The relatively low strength of AC-2, at 1.6 MPa, can be attributed to the low MCL of tobermorite. Previous studies [55,56] have reported that tobermorite acts as a microscopic framework supporting the pore structure, which plays a crucial role in terms of strength development.

After 2 h of a constant temperature, the internal and external temperatures of AC-3 reach 180 °C. At this point, CO_2_ is injected into the autoclave, which mitigates its impact on hydrothermal synthesis. In addition, the pore wall of the AC-3 carbonated zone is densely filled with calcium carbonate particles ranging in size from 1 to 5 μm in size (Figure 11b). The formation of dense layers of carbonation products [57] enhanced the strength.

After 4 h of a constant temperature, the high-alkalinity C–S–H, Ca(OH)_2_, AFt, and unhydrated CBSAC particles react with quartz to form tobermorite. At this time, injecting CO_2_ into the autoclave causes it to react with tobermorite to produce calcium carbonate and silica gel, as shown in Equation (12) [42].

(12) $$5CaO\cdot 6SiO_{2}\cdot 5H_{2}O+5CO_{2}\to 5CaCO_{3}+6SiO_{2}+5H_{2}O$$

In the carbonated region, the needle-like crystals are no longer tobermorite but silica-gels. This transformation is called “pseudomorphism”, and it originates from decomposed tobermorite [38,39]. Clusters of calcium carbonate form on the surface of the pseudomorphism, and the pore wall of the carbonated zone in AC-4 exhibits a relatively high density. In addition, the ^29^Si NMR results (Figure 12c and Table 7) indicate that introducing CO_2_ exerts a marginal effect on the MCL of the tobermorite. Consequently, the strength of AC-4 is 4.1 MPa, which exceeds that of AC-2 and AC-3 by 2.5 MPa and 0.8 MPa, respectively, demonstrating a considerable improvement (Figure 6b).

Compared with CBSAC AAC, the strength of autoclave–carbonation-cured CBSAC aerated concrete shows an alight decrease. However, it still meets regulatory standards, and its CO_2_ sequestration capability provides substantial benefits, making it a valuable option for promotion and application in relevant industries.

## 4. Prospects for Industrial Application

**Figure 15:**
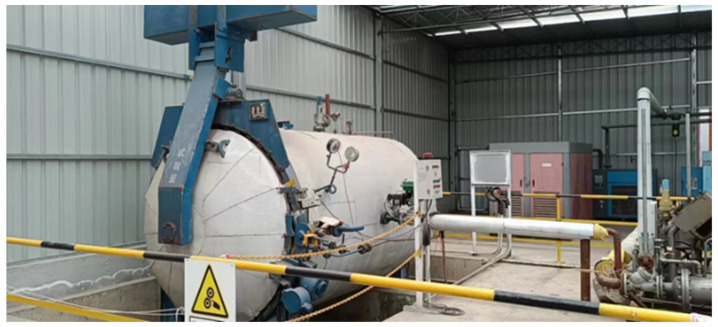
The 10 m^3^ autoclave equipment.

Figure 15 shows a 10 m^3^ autoclave equipment installed at the end of the cement rotary kiln to capture flue gas and superheated steam from waste heat boilers for conducting autoclave–carbonation curing. At present, waste heat boilers are set up at the head and tail of rotary cement kilns to recover and utilize waste heat from the flue gas [58]. After absorbing heat from the waste heat boiler, water becomes superheated steam, which can be imported into the autoclave to cure concrete. Additionally, the flue gas from waste heat boilers can be introduced into the autoclave through an air compressor, thereby enabling autoclave–carbonation curing of aerated concrete in cement plants.

As shown in Table 9, a comparative analysis is performed on product performance and economic feasibility between industrial autoclave and autoclave–carbonation curing of CBSAC aerated concrete. The data for autoclave curing are sourced from a CBSAC AAC production enterprise in Leshan, Sichuan, China. In autoclave–carbonation curing, the curing cost includes the cost of superheated steam generated by the waste heat boiler at the kiln tail and the power consumption related to air compressor usage for vacuum suction of flue gas at the kiln tail.

As shown in Table 9, the strength of industrially manufactured CBSAC AAC exceeds that of autoclave–carbonation-cured CBSAC aerated concrete, although it has a lower bulk density. This finding is consistent with laboratory study results. However, the curing cost for CBSAC AAC ranges from 32 to 39 CNY/m^3^, while the cost for autoclave–carbonation curing is considerably lower at 18.9 CNY/m^3^, resulting in a cost reduction of 13–20 CNY/m^3^.

Additionally, the CBSAC aerated concrete exhibits a carbon uptake of 40–60 kg/m^3^. According to the ratio specified in Section 2.2.1, 1 ton of CBSAC can produce 2.63 m^3^ of aerated concrete, which can absorb between 105 and 158 kg of CO_2_. The autoclave–carbonation curing aerated concrete lessens the environmental impact of the CBSAC industry and aligns with global efforts to combat climate change.

Currently, industrial implementation of autoclave–carbonation curing for CBSAC aerated concrete faces a challenge: the accumulation of saturated water vapor at the bottom and flue gas at the top of the autoclave. This situation often leads to ineffective CO_2_ uptake in the aerated concrete at the bottom, while the concrete at the top tends to exhibit lower strength.

## 5. Conclusions

This study investigated the effects of carbonation curing and autoclave–carbonation curing on the physical and mechanical properties, carbonation degree, pH value, hydration products, and pore characteristics of CBSAC aerated concrete. Based on laboratory research findings, autoclave–carbonation curing was tested utilizing a 10 m^3^ autoclave in cement plants. The conclusions of this study are as follows:

(1) During the autoclave curing process, the hydration products and unhydrated CBSAC particles react with quartz to form a highly polymerized plate-like tobermorite, which enhances the strength of CBSAC aerated concrete to 4.3 MPa;
(2) In autoclave–carbonation curing, delaying CO_2_ injection allows for better CO_2_ diffusion and reaction within the pores, increases the degree of carbonation from 19.1% to 55.1%, and the bulk density from 603.7 kg/m^3^ to 640.2 kg/m^3^. Furthermore, postponing the CO_2_ injection has little impact on the MCL of the tobermorite while leading to the formation of calcium carbonate clusters and needle-like silica gels in the carbonation zone, thereby contributing to a higher pore wall density and achieving a strength of 4.1 MP;
(3) The carbonation-cured samples contain products such as calcite, aragonite, C–S–H, and gypsum. While the carbonation reaction reduces the micropore volume of CBSAC aerated concrete, the limited amount of hydrates and carbonates formed does not effectively bond with sand particles. As a result, the compressive strength is only 0.8 MPa;
(4) Autoclave–carbonation curing presents promising prospects for industrial application. This methodology produces CBSAC aerated concrete, which exhibits a CO_2_ sequestration capacity of 40 to 60 kg/m^3^ and a compressive strength range of 3.6 to 4.2 MPa, meeting the strength requirements in the Chinese standard GB/T 11968 while reducing CO_2_ emissions from CBSAC factories.

## Figures and Tables

**Table 1:** Physical properties of CBSAC.

Physical Properties	Density (g/cm^3^)	Specific Surface Area (m^2^/kg)	45 µm Screen Residue Weight (%)	Setting Time (Min)	
				Initial	Final
CBSAC	3.03	365	12.7	32	55

**Table 2:** Chemical composition of raw materials (%).

Raw Materials	SiO_2_	Al_2_O_3_	Fe_2_O_3_	CaO	MgO	SO_3_	R_2_O	Loss
CBSAC	16.47	6.71	1.10	68.30	1.42	5.15	0.43	0.42
Red sand	71.54	14.89	4.42	1.37	1.49	1.50	2.70	2.09
Gypsum	1.30	0.73	0.12	31.5	1.10	45.0	-	20.25

**Table 3:** The particle distribution of raw material.

Raw Materials	D_10_ (μm)	D_50_ (μm)	D_90_ (μm)
Gypsum	1.9	14.5	76.0
Red sand	2.8	25.6	90.3
CBSAC	1.1	8.3	40.1

**Table 4:** Main mineral composition of CBSAC (%).

CaO	$C_{4}A_{3}\bar{S}$	C_2_S	CaSO_4_	Others
30.4	9.4	51.2	1.7	7.3

**Table 5:** Carbonation degrees of samples according to the curing regime.

Sample	Curing Regime	Average Carbonation Degree (%)	Standard Deviation
AC-1	Autoclave curing	0.0	/
AC-2	Autoclave–carbonation curing	19.1	2.3
AC-3		36.8	4.7
AC-4		55.1	6.2
AC-5	Carbonation curing	100.0	/

**Table 6:** pH values of samples.

Sample ID	AC-1	AC-2		AC-3		AC-4		AC-5
		(I)	(S)	(I)	(S)	(I)	(S)	
pH value	11.07	11.09	9.28	11.05	9.24	11.00	9.16	8.86

**Table 7:** Q values of samples with autoclave curing: AC-1, AC-2 (I), AC-3 (I), and AC-4 (I).

Samples	Q^1^	$Q_{P}^{2}$ (1Al)	$Q_{b}^{2}$	Q^3^ (1Al)	Q^3^ (0Al)	Q^4^	MCL
AC-1	10.5	13.6	32.8	21.9	5.8	15.3	40.6
AC-2 (I)	15.8	3.4	33.3	14.9	9.3	23.3	23.2
AC-3 (I)	12.4	12.9	34.3	14.9	8.7	16.7	27.6
AC-4 (I)	12.7	14.4	33.4	18.7	6.3	14.5	32.8

**Table 8:** Q values of samples with carbonation curing: AC-2 (S), AC-3 (S), AC-4 (S), and AC-5.

Samples	Q^1^	Q^2^	Q^3^	Q^4^
AC-2 (S)	5.1	5.7	22.7	66.5
AC-3 (S)	2.3	7.5	23.8	66.4
AC-4 (S)	5.0	18.4	16.9	59.6
AC-5	4.1	9.5	37.9	48.5

**Table 9:** Comparative analysis of product performance and economic feasibility.

Curing Method	Bulk Density (kg/m^3^)	Compressive Strength (MPa)	Curing Cost (CNY/m^3^)
Autoclave	540~560	4.4~4.9	32~39
Autoclave–carbonation	580~620	3.6~4.2	18.9

## Data Availability

Data will be made available upon reasonable request.

## List of Abbreviations

Abbreviations	Meaning
AAC	Autoclaved aerated concrete
OPC	Ordinary Portland cement
C_2_S	2 CaO·SiO_2_ (Belite)
C_3_S	3 CaO·SiO_2_
C_4_AF	4 CaO·Al_2_O_3_·Fe_2_O_3_
C_4_A_3_$\bar{S}$	Calcium sulfoaluminate: 4 CaO·3Al_2_O_3_·SO_3_ (Yeelimite)
AFt	Ettringite
C–S–H	Hydrated calcium silicate
C_2_SH	2CaO·SiO_2_·H_2_O
C_2_SH_2_	2CaO·SiO_2_·2H_2_O
f-CaO	Active calcium oxide (lime)
CBSAC	Calcium oxide–belite–calcium sulfoaluminate cementitious material
